# Resistance to Multiple Soil-Borne Pathogens of the Pacific Northwest, USA Is Colocated in a Wheat Recombinant Inbred Line Population

**DOI:** 10.1534/g3.116.038604

**Published:** 2017-02-01

**Authors:** Alison L. Thompson, Aaron K. Mahoney, Richard W. Smiley, Timothy C. Paulitz, Scot Hulbert, Kim Garland-Campbell

**Affiliations:** * Plant Physiology and Genetics Research Unit, United States Department of Agriculture-Agricultural Research Service, Maricopa, Arizona 85138; † Department of Plant Pathology, Washington State University, Pullman, Washington 99164; ‡ Columbia Basin Agricultural Research Center, Oregon State University, Pendleton, Oregon 97801; § Wheat Health, Genetics and Quality Research Unit, United States Department of Agriculture-Agricultural Research Service, Pullman, Washington 99164

**Keywords:** plant pathology, marker-assisted selection, breeding

## Abstract

Soil-borne pathogens of the Pacific Northwest decrease yields in both spring and winter wheat. Pathogens of economic importance include *Fusarium culmorum*, *Pratylenchus neglectus*, *P. thornei*, and *Rhizoctonia solani* AG8. Few options are available to growers to manage these pathogens and reduce yield loss, therefore the focus for breeding programs is on developing resistant wheat cultivars. A recombinant inbred line population, LouAu (MP-7, NSL 511036), was developed to identify quantitative trait loci (QTL) associated with resistance to *P. neglectus* and *P. thornei*. This same population was later suspected to be resistant to *F. culmorum* and *R. solani* AG8. This study confirms partial resistance to *F. culmorum* and *R. solani* AG8 is present in this population. Six major and 16 speculative QTL were identified across seven measured traits. Four of the six major QTL were found within the same genomic region of the 5A wheat chromosome suggesting shared gene(s) contribute to the resistance. These QTL will be useful in breeding programs looking to incorporate resistance to soil-borne pathogens in wheat cultivars.

Root and crown diseases caused by soil-borne pathogens decrease yields in both spring and winter wheat (*Triticum aestivum* L.) in the Pacific Northwest (PNW) of the United States. Pathogens of economic importance that are often found in complexes in the PNW include *Fusarium culmorum* (Wm. G. Sm.) Sacc., root-lesion nematodes *Pratylenchus neglectus* (Rensch 1924) Schuurmans and Stekhoven 1941 and *P. thornei* Sher and Allen 1953, and *Rhizoctonia solani* AG8 Kühn (Mahoney *et al.* 2016; Paulitz 2006; Paulitz *et al.* 2002; Smiley *et al.* 2005a,b; Smiley and Patterson 1996).

*Fusarium* crown rot is caused by a complex of fungal species of which *F. culmorum* and *F. pseudograminearum* (O’Donnell and Aoki) (= *F. graminearum* group I, = *Gibberella coronicola*) are the most significant. Symptoms include rotted plant roots, crowns, and lower stems causing browning of tissues. Crown rot infection reduces plant biomass due to restricted intake of water and nutrients. As a result, prior to harvest, the developing grain in the spike dies, causing whiteheads at the onset of seed development (Paulitz *et al.* 2002; Smiley *et al.* 2005c). Surveys have found *F. culmorum* isolates in as many as 36% of sampled fields in the PNW states of Washington and Oregon (Poole *et al.* 2013; Smiley and Patterson 1996). Potential yield loss by *Fusarium* crown rot in PNW winter wheat has been estimated at 35% (Smiley *et al.* 2005c).

Root-lesion nematodes (RLN) *P. neglectus* and *P. thornei* feed on root cells, causing brown lesions, and reduced root and shoot biomass (Townshend *et al.* 1989; Zunke 1990). The damage to the roots limits water and nutrient uptake from the soil, resulting in reduced grain quality and yield (J. P. Thompson *et al.* 1999, 2008). Both RLN species have been detected in 95–96% of sampled fields in the PNW and Intermountain west regions of the United States (Smiley *et al.* 2004; Strausbaugh *et al.* 2004). Yield reduction in the PNW has been reported as high as 60% for *P. thornei*, and 35% for *P. neglectus* (Smiley *et al.* 2005a,b).

Rhizoctonia root rot and bare patch disease are common in notill farming systems (Cook *et al.* 2002; Paulitz *et al.* 2002), a practice that has increased steadily in the PNW, ranging from 11 to 70% of planted acreage across PNW counties (Young 2009). Fungal infection causes rotting of the seminal and crown root tissue, resulting in brown lesions, spear tipping of roots, and stunted seedlings (Paulitz *et al.* 2002; Weller *et al.* 1986). In the field, *R. solani* AG8 causes Rhizoctonia bare patch disease, characterized by large circular dead patches of wheat. Yield losses by *Rhizoctonia* have been reported between 10 and 30%, and are highly correlated with disease severity (Cook *et al.* 2002; Mahoney *et al.* 2016; Paulitz *et al.* 2002).

Cultural methods to reduce soil-borne diseases include tillage, reducing nitrogen fertilizer, or rotation to less profitable crops, all of which are used, but not favored, in dryland wheat farming systems. *Fusarium* crown rot damage can be reduced by delayed planting and management of nitrogen fertilizer applications (Paulitz *et al.* 2002). Rhizoctonia root rot can be reduced through tillage, and managing weeds and volunteer wheat well before planting (Gill *et al.* 2002; Paulitz *et al.* 2002; Smiley *et al.* 1992). RLNs have been more difficult to manage, they have an extensive host range, reducing the effectiveness of crop rotations, and can be found as low as 90 cm in soil profiles, essentially evading tillage disturbance (Smiley *et al.* 2008; Smiley and Machado 2009). Therefore, the focus for management of these diseases is on developing resistant and/or tolerant wheat cultivars.

The search for resistance to these soil-borne pathogens has been challenging. Phenotyping is time-consuming and difficult, and subject to spatial and environmental variation in the field (Collard *et al.* 2005; Schmidt *et al.* 2005; Schneebeli *et al.* 2016; Sharma *et al.* 2011). Often, the identified resistant or partial-resistant germplasm have been wild relatives, landraces, and synthetic hexaploids that also possess nondesirable traits (Li *et al.* 2010; Mahoney *et al.* 2016; Thompson *et al.* 1999, 2016). To improve breeding efficiency, it is desirable to define the genetic architecture (number and location of loci) for resistance to these pathogens. The LouAu recombinant inbred line (RIL) population was developed by Thompson *et al.* (2015). The resistant parent IWA8608077 (PI621458), an Iranian landrace, was previously identified as resistant to both *Pratylenchus* species by Sheedy *et al.* (2007) and A. L. Thompson *et al.* (2008). The LouAu population was phenotyped for resistance to *P. neglectus* and *P. thornei*, but the whole population has not yet been phenotyped for resistance to other soil-borne pathogens.

The objectives of this study were to (i) phenotype the LouAu RIL population for response to *F. culmorum* and *R. solani* AG8; and (ii) identify quantitative trait loci (QTL) associated with reduced infection to *F. culmorum*, both *Pratylenchus* species, and *R. solani* AG8.

## Materials and Methods

### Plant material and genotyping

The LouAu RIL population (MP-7, NSL 511036) was derived by crossing the PNW cultivar “Louise” (PI634865) with the Iranian landrace IWA8608077 (PI621458). Louise is a soft white spring wheat cultivar, with good milling and baking characteristics (Kidwell *et al.* 2006), and high-temperature adult plant resistance to stripe rust (Carter *et al.* 2009). IWA8608077 is a late-maturing hexaploid, and a member of an 11,000 accession collection that has been phenotyped for resistance or tolerance to many biotic stresses (Thompson *et al.* 2016). The development, genotyping, and linkage map of the LouAu RIL population was previously described by Thompson *et al.* (2015). Briefly, the population was genotyped at F_5_:F_6_ with simple sequence repeat markers, and also with SNP markers using the Illumina wheat 9K SNP chip (Cavanagh *et al.* 2013). The population was also genotyped for known major developmental genes. Linkage groups were generated using JoinMap software (Kyazma B.V., The Netherlands). Linkage groups were assigned to genome-specific chromosomes by comparison to the wheat 9K SNP consensus map (Cavanagh *et al.* 2013). The linkage map contains 2038 markers mapped to 26 linkage groups, representing all 21 of the *T. aestivum* chromosomes. The total map distance is 2426.8 cM, with an average intermarker spacing at 1.33 cM.

### Greenhouse plant disease phenotyping

The population was phenotyped for response to multiple soil-borne pathogens at generations F_5_–F_8_ as described below. Screening for response to soil-borne pathogens can be time-consuming and variable (Schneebeli *et al.* 2016; Sharma *et al.* 2011; Smiley and Patterson 1996); therefore, the phenotyping assays included several susceptible and resistant checks (Table 1).

**Table 1:** List of checks for the greenhouse assays

Entry Name	Check response				Origin	Germplasm Type	Identification Number	Citation
	*F. culmorum*	*P. neglectus*	*P. thornei*	*R. solani* AG8				
Alpowa	S*^a^*	S	S		PNW, US	Cultivar	PI566596	Sheedy *et al.* (2007)
CPI133872		R	R		CIMMYT	Synthetic	CIGM89.576	Zwart *et al.* (2005)
Excalibur		R			Australia	Cultivar	AUS99161	Williams *et al.* (2002)
Gatcher			S		Australia	Cultivar	AUS99013	Thompson *et al.* (1999)
GS50a			R		Australia	Breeding line	n/a	Thompson *et al.* (1999)
IDO377s		S	S		PNW, US	Cultivar	PI642378	Sheedy *et al.* (2007)
Iraq 43			R		Middle East	Landrace	AUS4926	Schmidt *et al.* (2005)
IWA8608077*^b^*		R	R		Middle East	Landrace	PI623470	Sheedy *et al.* (2007) and A. L. Thompson *et al.* (2008)
Janz		S	S		Australia	Cultivar	AUS24794	Zwart *et al.* (2005)
Louise*^b^*	S*^a^*	S	S	S	PNW, US	Cultivar	PI634865	Sheedy *et al.* (2007) and Mahoney *et al.* (2016)
Macon	S*^a^*				PNW, US	Cultivar	PI617072	
McNeal		S	S		PNW, US	Cultivar	PI574642	Sheedy *et al.* (2007)
Morocco 426			R		Middle East	Landrace	AUS13124	Schmidt *et al.* (2005)
Otis	S*^a^*	S	S		PNW, US	Cultivar	PI634866	Sheedy *et al.* (2007)
Persia 20		R			Middle East	Landrace	CI 11283	Sheedy *et al.* (2007)
Seri (M82)			S		CIMMYT	Cultivar	CM33027	Sheedy *et al.* (2007)
Sunco	R*^a^*				Australia	Cultivar	AUS99130	

Each entry is identified with the corresponding response to *F. culmorum*, both *Pratylenchus* sp. and *Rhizoctonia solani* AG8, the origin of the entry, and the identification or registration number for each. The citations are given for each entry where the response was identified. S, susceptible check; R, resistant check.

a Response is from Y. Manning-Thompson, unpublished data.

b Parent of the LouAu mapping population.

#### Fusarium culmorum:

Inoculum was prepared following the procedure of Smiley *et al.* (2005c) with twice-autoclaved millet seed. Five PNW native isolates of *F. culmorum* (F70110023, F70110097, F70110022, F70110019, and F70110126) collected from a survey in 2011 (T.C. Paulitz, unpublished data) were maintained on either 0.5-strength potato dextrose agar (Sigma-Aldrich) or water agar (Sigma-Aldrich). Each isolate was individually seeded onto the autoclaved millet in 4 l Erlenmeyer flasks and kept at 22–24° in the dark for 3–4 wk. After 1 wk, flasks were shaken every week to ensure even colonization of the millet seed. Colonized millet seed was broken up to remove clumps, air dried, and stored at room temperature (22–24°) in paper bags. Isolates were then blended in equal proportions before application in the greenhouse assays.

The LouAu RILs, parent lines, and checks (Table 1) were evaluated in two experiments with five replicates each in a randomized block design. All plants were grown in D40H Deepots (Stuewe and Sons, Oregon) with Sunshine Mix#4 potting soil (Sungro, Canada), and Osmocote 14-14-14 slow release fertilizer (Scotts Co. LLC) as described by Poole *et al.* (2012) for growth room assays. The experiments were conducted in the greenhouses at the Washington State University Plant Growth Facility (WSU-PGF) in Pullman, WA. Temperatures were maintained at 22–24° with 12-hr day length supplemental lighting. Two-wk-old seedlings were inoculated with ∼1 g of the blended colonized millet at the base of the seedling, then covered with 1 g of potting soil. Plants were evaluated for *F. culmorum* symptoms 12 wk after infection. *F. culmorum* infection was determined by visually scoring the crown and internodes for browning using a 0–10 rating system, where 0 = no disease, and 10 = dead plant with severe symptoms, as described by Poole *et al.* (2012). The rating for these symptoms will be referred to as Fc-Score for the remainder of the paper.

#### Pratylenchus species:

Inoculum was prepared following a modified monoxenic carrot culture protocol described by Castillo *et al.* (1995). Prepared carrot disks were inoculated with a single nematode, and kept in a dark growth cabinet (Percival Scientific) at 22° for at least 2 months prior to extraction in water. Nematodes were collected from a 2010 survey of dryland wheat fields in eastern WA (Kandel *et al.* 2013), and maintained in water until carrot disk inoculation. *Pratylenchus* inoculum for each species was quantified using a nematode counting slide (Chalex Corporation) with 1.5 ml aliquots, and species-specific primers developed by Yan *et al.* (2008) were used to identify *P. neglectus* or *P. thornei*.

The LouAu RILs, parent lines, and checks (Table 1) were evaluated in separate experiments for each *Pratylenchus* species with two replicates each in randomized block designs. All plants were grown in D40H Deepots with 150 g of 3:1 pasteurized Palouse silt loam soil to sand and Osmocote 14-14-14 slow release fertilizer (Scotts Co. LLC) as described by Thompson *et al.* (2016). All nematode experiments were conducted in a Conviron GR48 (Controlled Environments LTD) chamber at the WSU-PGF. Temperatures were maintained at 24° with 14-hr day lengths. Two-wk-old seedlings were inoculated with ∼100 nematodes (*P. neglectus* or *P. thornei*) in water at the base of the seedling, then covered with ∼1 g of the 3:1 soil mixture. Plants were evaluated for *Pratylenchus* damage 12 wk after inoculation. *Pratylenchus* damage was determined by visually scoring root browning on a rating scale of 1–5, where 1 = little to no root browning, and 5 = complete root browning (Thompson *et al.* 2016). The rating for these symptoms will be referred to as Pn-Score or Pt-Score for *P. neglectus* or *P. thornei*, respectively for the remainder of the paper. After damage was assessed, soil and root samples were collected in plastic bags, chilled to 4° overnight and sent to Western Laboratories Inc. (Parma, ID; http://www.westernlaboratories.com) for nematode extraction using a modified Oosterbrink elutriator (Smiley *et al.* 2011), followed by enumeration with a Chalex counting slide. The protocol followed by Western Laboratories concentrates the final extracted volume of nematodes to ∼10 ml of water, and a single 1 ml aliquot is taken for nematode enumeration. The total count for this assay will be referred to as Pn-Count or Pt-Count for *P. neglectus* or *P. thornei*, respectively for the remainder of the paper.

#### Rhizoctonia solani AG8:

Inoculum was prepared following the procedure by Paulitz and Schroeder (2005) with autoclaved oat seed. A *R. solani* AG8 isolate (isolate C1, Weller *et al.* 1986) was grown on water agar, and plugs were used to seed the autoclaved oats in Erlenmeyer flasks. Flasks were kept in the dark at 22–24° for 3–4 wk, then dried for 2 d in a laminar flow hood. Colonized seeds were ground with a coffee grinder, and passed through a 1-mm and 250-µm sieve. Ground oat particles were collected between the two sieve sizes. Dilution plating was used to quantify inoculum using *Rhizoctonia* selective media (Paulitz and Schroeder 2005).

The LouAu RILs and parent lines were evaluated in three experiments, with three replicates each, in a completely randomized design. All experiments were conducted in a Conviron GR48 (Controlled Environments LTD) at WSU-PGF at 15–18° for 14 d, with a 12-hr day length. To synchronize germination, seeds of each RIL and parent line were cold stratified for 24 hr at 4° on premoistened paper, then germinated in the dark for 24 hr at 22–24°. All germinated seeds were transplanted to D27L Deepots (Stuewe and Sons, Oregon) in each of two treatments; with either ∼60 g pasteurized Ritzville silt loam soil inoculated with colonized ground oats at 200 propagules per gram of soil, or ∼60 g pasteurized Ritzville silt loam soil with sterile ground oats. Seeds in each treatment were covered with noninoculated soil. Seedlings were evaluated for *Rhizoctonia* symptoms 2 wk after transplanting. Previous experiments had indicated that seedling stunting by *Rhizoctonia* infection is a good indicator of resistance or tolerance at the seedling stage (Mahoney *et al.* 2016). Seedlings were measured from the crown to the top of the first and tallest leaf lengths. Because leaf length varied according to genotype, leaf length was measured as a percent reduction from the control (noninoculated) plant, by comparing the first and tallest leaf of each RIL. The leaf length reduction formula was *y* = (*a* − *b*)/*a* × 100, where *y* = percent leaf length reduction; *a* = the leaf length of the noninoculated plant; and *b* = the leaf length of the corresponding inoculated plant. The calculated percent reduction will be referred to as Fl-Stunt, or Tl-Stunt, for first leaf and tallest leaf reductions, respectively, for the remainder of the paper.

### Statistical analysis

The SAS software MIXED procedure (SAS Institute Inc 2012) was used to fit a linear model to each trait for outlier removal and least squared means (lsmeans) estimates; the models for each trait are below. The data for each assessed trait was first examined for significant outliers that could influence an estimated mean. Outliers were determined by setting an upper and lower limit for the studentized deleted residuals calculated from the population size with a criterion of *α* = 0.05 (Kutner *et al.* 2004). After outliers were removed, the lsmeans were estimated across experiments for each RIL for each trait assessed.

#### Fusarium culmorum:

The model for analysis of the *F. culmorum* infection symptoms (Fc-Score) was:

$$Y_{ijkl}=µ+{\text{entry}}_{i}+{\text{exp}}_{j}+\text{rep}{\left(\text{exp}\right)}_{jk}+\text{tray}{\left(\text{rep}*\exp\right)}_{jkl}+{\left(\text{entry}\times \text{exp}\right)}_{ij}+\varepsilon_{ijkl}$$

where *Y_ijkl_* is the Fc-Score of an individual Deepot; *entry_i_* is the fixed effect of the *i*th entry (RIL or check under evaluation); *exp_j_* is the fixed effect of the *j*th experiment; *rep*(*exp*)*_jk_* is the random effect of the *k*th replication nested within the *i*th experiment; *tray*(rep*exp)*_jkl_* is the random effect of the *l*th tray (vessel in which Deepots were held) nested within the *k*th replication within the *j*th experiment; (*entry* × *exp*)*_ij_* is the fixed effect of the of the interaction between the *i*th entry and the *j*th experiment; and *ε_ijkl_* is the error term.

#### Pratylenchus species:

The statistical model for analysis of the nematode root damage (Pn-Score or Pt-Score) was:

$$Y_{ijkl}=µ+{\text{entry}}_{i}+{\text{trt}}_{j}+\text{rep}{\left(\text{trt}\right)}_{jk}+\text{tray}{\left(\text{rep}*\text{trt}\right)}_{jkl}+{\left(\text{entry}*\text{trt}\right)}_{ij}+\varepsilon_{ijkl}$$

where *Y_ijkl_* is the Pn-Score or Pt-Score of individual Deepot; *entry_i_* is the fixed effect of the *i*th entry; *trt_j_* is the fixed effect of the *j*th *Pratylenchus* treatment (either *P. neglectus* or *P. thornei*); *rep*(*trt*)*_jk_* is the random effect of the *k*th replication nested within the *j*th treatment; *tray*(rep*trt)*_jkl_* is the random effect of the *l*th tray nested within the *k*th replication within the *j*th treatment; and *ε_ijkl_* is the error term.

The statistical model for analysis of the total nematode counts (Pn-Count or Pt-Count) was:

$$f{\left(y\right)}_{\text{ijkl}}=µ+{\text{entry}}_{i}+{\text{trt}}_{j}+\text{rep}{\left(\text{trt}\right)}_{jk}+\text{tray}{\left(\text{rep}*\text{trt}\right)}_{jkl}+{\left(\text{entry}*\text{trt}\right)}_{ij}+\varepsilon_{ijkl}$$

where *ƒ*(*y*)*_ijkl_* is the log transformation of Pn-Count or Pt-Count of an individual Deepot; *entry_i_* is the fixed effect of the *i*th entry; *trt_j_* is the fixed effect of the *j*th *Pratylenchus* treatment (either *P. neglectus* or *P. thornei*); *rep*(*trt*)*_jk_* is the random effect of the *k*th replication nested within the *j*th treatment; *tray*(rep*trt)*_jkl_* is the random effect of the *l*th tray nested within the *k*th replication within the *j*th treatment; and *ε_ijkl_* is the error term. The log transformation was used because the *Pratylenchus* count data were highly right skewed so a natural logarithmic transformation ln *x* + 1 (*x* = count) was applied before data were analyzed.

#### Rhizoctonia solani AG8:

The model for analysis of the *R. solani* infection symptoms (Fl-Stunt or Tl-Stunt) was:

$$Y_{ij}=µ+{\text{entry}}_{i}+{\text{exp}}_{j}+\varepsilon_{ij}$$

where *Y_ij_* is the calculated Fl-Stunt or Tl-Stunt of an individual Deepot; *entry_i_* is the fixed effect of the *i*th entry; *exp_j_* is the fixed effect of the *j*th experiment; and *ε_ij_* is the error term. Due to the way in which *Rhizoctonia* symptoms were calculated (noninoculated minus inoculated measurements), the tray and replication effects became collapsed within the experiment and could not be separated for statistical analysis. The collapse of terms also limited the available degrees of freedom, and so an interaction effect between entry and experiment could not be determined.

To determine if the assessed traits were related, correlation coefficients were determined using the lsmeans from the analyses described above. Pearson’s *r* and calculated *P*-value were calculated using the SAS software CORR procedure. Correlations were considered significant at probability *P* ≤ 0.05. The heritability [*h^2^* = Var(*G*)/Var(*P*)] for each trait on a family-mean basis was estimated for each trait using the LouAu RILs only; the parent lines and checks were removed from the data sets. The statistical models for each trait were the same as described above except that each term in the model was considered random. The asycov option in the SAS software MIXED procedure (Self and Liang 1987) was used to calculate heritability with the SAS code provided by Holland *et al.* (2003).

The lsmeans for each trait were used to identify QTL with inclusive composite interval mapping (ICIM) (Li *et al.* 2007) for biparental populations with the ICIM-ADD function in QTL IciMapping v4.1.0 software (http://www.isbreeding.net). Seven markers were dropped from the linkage maps due to colocalization. Mapping parameters were set to stepwise regression with a walk interval of 1 cM. Significance thresholds were calculated by permutation (1000 permutations at *P* < 0.05) for major QTL. To detect speculative QTL the significance threshold was changed to manual input setting with LOD score to 1.5. Where QTL were detected, the percent variation explained by the QTL and additive effect were also calculated.

### Data availability

Lsmeans from the linear models for all soil-borne pathogen traits used in QTL analysis are in Supplemental Material, File S1. Linkage map information for the LouAu population is available in File S2. Remaining genotypic data for the 150 RILs are available in File S3.

## Results

### Greenhouse plant disease phenotyping

**Figure 1:**
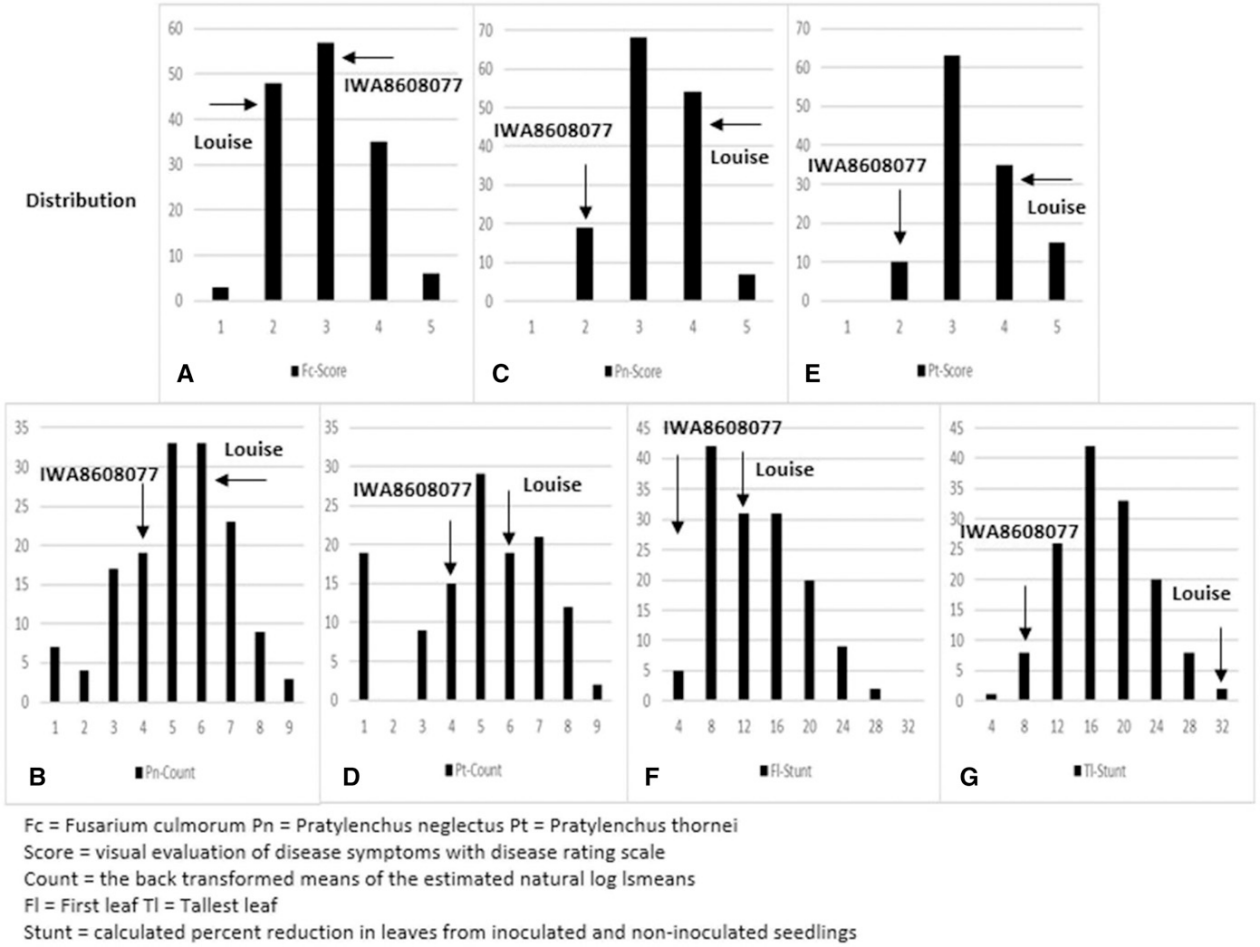
Histograms showing the distribution for the seven soil-borne pathogens assessed in the greenhouse assays using the lsmean estimates. (A) Fc-score, (B) Pn-count, (C) Pn-Score, (D) Pt-Count, (E) Pt-Score, (F) Fl-Sunt, and (G) Tl-Stunt.

The root disease symptoms caused by *F. culmorum* were moderate, and there was not a significant difference in response between the two parents. Nevertheless, there was a significantly different distribution of response to *F. culmorum* among the RILs (Figure 1A). The *P. neglectus* counts (Pn-Count) in both experiments were lower than *P. thornei* (Pt-Count) when averaged over the RIL population (Table 2). Both Pn-Count and Pt-Count showed a normal distribution after total count data were log transformed in the RIL population (Figure 1, B and D). The root damage due to nematode infection (Pn-Score and Pt-Score) were lower in IWA8608077 than Louise, as expected, and showed a similar range in the RIL population for both *Pratylenchus* species (Table 2). The Pn-Score distribution followed a normal curve, while the Pt-Score is skewed right toward increased root damage (Figure 1, C and E). The difference in Pt-Count and Pt-Score distributions indicate *P. thornei* caused more root damage than *P. neglectus*. Similar to the *Pratylenchus* traits, the *Rhizoctonia* symptoms were lower in the IWA8608077 parent than the Louise parent (Table 2). The distribution for first leaf stunting (Fl-Stunt) was skewed left toward decreased leaf reduction (Figure 1F). The range for tallest leaf stunting (Tl-Stunt) was slightly bigger than Fl-Stunt (Table 2), and showed a normal distribution in the RIL population (Figure 1G).

**Table 2:** Simple statistics from calculated lsmeans for the parent lines and the LouAu RIL population and estimated family-mean heritability for each trait measured

	IWA8608077		Louise		LouAu RILs				Heritability	
Trait	lsmean	SD	lsmean	SD	lsmean	SD	Minimum	Maximum	h2	SE
Fc-Score	3.1	2.69	2.8	2.20	2.4	0.80	0.8	4.4	23.7	0.13
Pn-Count	121.3	0.01	209.2	1.18	107.0	1.78	1.2	4600.4	50.8	0.13
Pn-Score	2.0	0.00	4.0	0.00	2.9	0.73	1.3	5.2	54.8	0.11
Pt-Count	62.0	0.01	740.8	0.61	89.3	2.21	1.9	8748.2	46.7	0.14
Pt-Score	2.0	0.00	4.0	0.01	3.0	0.87	1.3	5.3	85.5	0.03
Fl-Stunt	0.9	0.77	13.6	6.50	11.5	5.39	2.3	26.8	33.3	0.10
Tl-Stunt	9.2	6.25	34.1	12.64	15.8	5.37	3.0	29.6	11.4	0.07

SD, standard deviation from the calculated mean; Fc, *F*. *culmorum*; Pn, *P. neglectus*; Pt, *P. thornei*; Score, visual evaluation of disease symptoms with disease rating scale; Count, the back transformed means of the estimated natural log lsmeans; Fl, First leaf; Tl, Tallest leaf (from Rhizoctonia solani AG8 experiments); Stunt, calculated percent reduction in leaves from inoculated and noninoculated seedlings.

Of the possible correlations between all seven traits, only five were found to be significant at α ≤ 0.05. The Fc-Score trait was significantly related to three of the seven traits measured, although correlation values were low (<30%; Table 3). Estimated heritability for the traits ranged from 11.4 to 85.5% (Table 2). The estimated Fc-Score heritability is somewhat low compared to other *Fusarium* greenhouse assays reported by Poole *et al.* (2012), but is not out of range for soil-borne pathogen traits. The Pn-Count and Pt-Count heritability estimates (Table 2) fall within those previously reported by Thompson *et al.* (2012) and Sheedy *et al.* (2012) using total count data. The Pn-Score and Pt-Score estimates were higher than those for the count data (Table 2) indicating this is a more consistent phenotype to measure plant-nematode interactions. The estimated heritability for *R. solani* AG8 was lower than previously reported by Mahoney *et al.* (2016), which were calculated using shoot length reduction.

**Table 3:** Calculated Pearson correlation coefficients (*r*) and associated *P*-values for each trait measured in the LouAu population

	Fc-Score	Pn-Count	Pn-Score	Pt-Count	Pt-Score	Fl-Stunt	Tl-Stunt
Fc-Score	1.000	0.051	0.280*	0.105	0.216*	−0.017	0.293*
	0.001	0.578	0.002*	0.250	0.017*	0.855	0.001*
Pn-Count		1.000	0.055	0.074	0.034	0.071	0.004
		0.001	0.548	0.419	0.712	0.438	0.962
Pn-Score			1.000	0.129	0.382*	0.032	0.032
			0.001	0.159	<0.0001*	0.726	0.725
Pt-Count				1.000	0.057	−0.007	0.188*
				0.001	0.536	0.936	0.039*
Pt-Score					1.000	0.140	0.056
					0.001	0.127	0.542
Fl-Stunt						1.000	0.058
						0.001	0.525
Tl-Stunt							1.000
							0.001

* *P*-values significant when α ≤ 0.05.

### QTL analysis

The calculated significance threshold by permutation (1000 permutations at *P* *<* 0.05) was 3.1842. A total of six QTL with LOD > 3.0 were found across the seven traits, and 16 QTL with LOD < 3.0 (Table 4 and File S4). These 22 QTL were found on 11 of the 21 wheat chromosomes, with LOD scores ranging from 1.51 to 9.40. The Pn-Count trait had the greatest number of QTL identified (7) over five chromosomes, and had the QTL with the highest LOD score. Only speculative QTL (LOD < 3.0) were found for traits Pt-Count and Fl-Stunt. The percent variation explained by the identified QTL ranged from 2.5 to 17.1%. The QTL marker spacing was <10.0 cM for 17 of the 22 identified QTL indicating the marker coverage, and linkage maps were sufficient to detect important resistance loci.

**Table 4:** Trait identified QTL using ICIM program in the LouAu RIL mapping population, and calculated lsmeans from greenhouse experiments

Trait	QTLName	Chrm	QTL Pos. (cM)	Left Marker	Left Pos.	Right Marker	Right Pos.	LOD	PVE (%)	ADD	Allele (Parent)
Fc-Score	QFs.wsu-5A.2	5AL.2	116.0	IWA4046	115.5	IWA7878	116.5	4.13	12.2	−0.28	IWA8608077
Pn-Count	QRlnn.wsu-2Aa	2AL	78.0	IWA6874	77.5	IWA4627	78.5	5.98	9.2	−0.72	Louise
Pn-Count	QRlnn.wsu-2Ab	2AL	40.0	IWA8424	36.5	IWA3193	42.5	9.40	16.6	−0.97	IWA8608077
Pn-Score	QRlnn.wsu-5A.2	5AL.2	112.0	IWA2743	111.5	IWA3363	112.5	6.03	14.5	−0.31	IWA8608077
Pt-Score	QRlnt.wsu-5A.2	5AS.2	79.0	barc319	74.5	IWA3702	84.5	5.00	17.1	−0.37	IWA8608077
Tl-Stunt	QRs.wsu-5A.2	5AL.2	112.0	IWA2743	111.5	IWA3363	112.5	3.67	11.5	−1.88	IWA8608077

PVE, percent variation explained by the QTL for the designated trait; ADD, additive effect of the identified allele for the QTL.

The 5A chromosome was the location of six QTL associated with decreased soil-borne pathogen symptoms. The 5A QTL identified for Pn-Score (QRlnn.wsu-5A.2) and Tl-Stunt (QRs.wsu-5A.2) share the same cM position, and map within 4 cM of the Fc-Score QTL (QFs.wsu-5A.2) (Table 4). These QTL map within 33 cM of the centromere, closer than the Pt-Score 5A QTL (QRlnt.wsu-5A.2). The allele (A) with reduced disease symptoms came from the IWA8608077 parent for all four of these QTL (Table 4). These same traits also possessed significant correlations with each other (Table 3) indicating shared resistance gene(s) coming from the IWA8608077 parent. Sequence for the significant SNP markers can be found in File S5.

Three QTL were identified on chromosome 2A that were within 42 cM marker spacing to each other (QRlnn.wsu-2Aa, QRlnn.wsu-2Ab, and QRs.wsu-2A) (Table 4 and File S4). The Pn-Count and Fl-Stunt traits were not significantly correlated (Table 3), but this 2A genome region could contain gene(s) associated with resistance to all three pathogens. Two of these three QTL showed the allele (B) with reduced disease symptoms was derived from the Louise parent. Louise was also the source of the allele associated with reduced disease symptoms for 12 of the 16 speculative QTL identified (File S4) suggesting transgressive segregation in this population.

## Discussion

The LouAu RIL population was initially developed to identify QTL associated with resistance to *P. neglectus* and *P. thornei*. Differences in root architecture traits between the IWA8608077 and Louise parents indicated this population may also be useful as sources of resistance or partial-resistance to other soil-borne pathogens. Particularly interesting was the significant increase of root lignin content in the IWA8608077 parent compared to the Louise parent (Thompson *et al.* 2015), and the distribution to the outermost root cells (A.L. Thompson, unpublished data). Increased lignification has been associated with decreased appressorium penetration by fungal species in grasses, including wheat (Vance *et al.* 1980), and failure to penetrate wheat leaves by the foliar fungal pathogen *Botrytis cinerea* (Ride and Pearce 1979). Since IWA8608077 was found to be resistant to the *Pratylenchus* species, *F*. *culmorum*, and *R. solani* AG8, it is possible the increased root lignin is contributing to the resistance, and would be an excellent breeding target for multi-pathogen resistance introgression.

Heritability estimates for three of the seven traits evaluated were below 50%, which is common in soil-borne diseases. These traits are difficult to phenotype, which increases environmental variation, limiting the ability to accurately estimate genetic effects. The low heritability calculated for *F. culmorum* in this study could be because of low phenotypic variation, or the scoring method used to quantify disease symptoms. The 0–10 scale used to rate *F. culmorum* symptoms was developed for *F. pseudograminearum* (Poole *et al.* 2012), yet *F. culmorum* shows more root browning and fewer stem symptoms than *F. pseudograminearum* (Smiley *et al.* 2005c). The low heritability estimates for the *R. solani* AG8 traits Fl-Stunt and Tl-Stunt is most likely due to the calculated percent reduction used to measure stunting for these two traits. The random experimental variation was confounded with the experiment model term, limiting the ability to quantify error, and therefore estimate the genetic effects.

Chromosome 5A had four major QTL associated with reduced disease incidence to multiple different soil-borne pathogens in this study. Resistance QTL have not previously been identified on chromosome 5A for *F. culmorum*, *R. solani* or either *Pratylenchus* species. A QTL for resistance to *P. neglectus* was identified on barley chromosome 5H, which shares synteny with wheat chromosomes group 5 (Sharma *et al.* 2011). The 5A chromosome region that contributed resistance to *F. culmorum* and *R. solani*, also decreased damage from *P. neglectus*, and probably accounted for the correlation between these three traits in the RILs. It is therefore possible that all three traits are controlled by the same gene, or closely linked genes, which may confer a nonspecific defense to soil-borne pathogens.

For many of the speculative QTL identified in this study, the allele with reduced disease symptoms was derived from Louise, which was considered to be the susceptible parent. Transgressive segregation has been shown to occur in wide crosses, and can be attributed to the complementary action of genes from the two parents, or the unmasking of recessive genes in the wild parent (deVicente and Tanksley 1993). For this study, the genes within the speculative QTL regions could be acting complementary to resistance genes from the IWA8608077 parent but further study is needed. Increased root lignin content is suspected to be involved as a potential defense mechanism contributing to IWA8608077 resistance to soil-borne pathogens. The lignin biosynthesis pathway is highly conserved throughout the plant kingdom as it is a major contributor to plant cell walls (Cosgrove 2001). It would be of great interest to determine if the QTL found in this study contain genes associated with the lignin biosynthesis pathway, and if alleles associated with resistance increase lignin content.

The incorporation of landrace material into breeding programs for genetic and trait diversity has become of great interest, as concerns of narrow genetic pools in wheat breeding programs increase (Able *et al.* 2006; Hoisington *et al.* 1999; Reif *et al.* 2005; Warburton *et al.* 2006). Landraces have been shown to be sources for resistance and tolerance to complex traits (Ehdaie *et al.* 1988; Jafari-Shaberstari *et al.* 1995; Waines and Ehdaie 2007), and so are candidates for mapping population development. Difficulties that can be associated with the use of landraces in breeding programs emphasize the importance of identifying cost effective tools, like marker assisted selection, for resistance introgression and to reduce linkage drag. In this study, useful markers were identified for multiple soil-borne pathogen traits from a single landrace parent.

## Supplementary Material

Supplemental material is available online at www.g3journal.org/lookup/suppl/doi:10.1534/g3.116.038604/-/DC1.
